# Supplementary material for: Evaluation capacity building in a rural Victorian community service organisation: A formative evaluation
Source: PLoS One. 2025 Jul 30;20(7):e0322906. doi: 10.1371/journal.pone.0322906 (PMC12310013; doi:10.1371/journal.pone.0322906)
Supplement: S1 Table — (DOCX) [file pone.0322906.s001.docx]

*Supplementary Material 1. Dimension and explanation of RE-AIM framework (39).*

| **Dimension** | **Explanation** |
| --- | --- |
| Reach | Absolute number or proportion of people who were approached by intervention communication and are possibly interested in participating |
| Effectiveness | Impact that the intervention has on the desired outcomes |
| Adoption | Absolute number, proportion, or rate of people or settings who are willing to implement the intervention |
| Implementation | Fidelity of the intervention to the protocol, as well as the consistency of delivery as intended, time*, and cost* of the intervention |
| Maintenance | Degree to which an intervention becomes institutionalised and part of the policy and practices within an organisation |

*Note: *Time and cost have been excluded from this evaluation.*
