# Supplementary material for: Evaluation capacity building in a rural Victorian community service organisation: A formative evaluation
Source: PLoS One. 2025 Jul 30;20(7):e0322906. doi: 10.1371/journal.pone.0322906 (PMC12310013; doi:10.1371/journal.pone.0322906)
Supplement: S1 File — (DOCX) [file pone.0322906.s002.docx]

*Supplementary Material 2. Baseline and follow-up questionnaires.*

**Demographics**

- Length in role *(less than one year, between one and two years, between two and five years, more than five years)*
- Level of position *(executive manager, manager, team or practice lead, practitioner, other)*
- Employment status *(full time, part time, casual)*
- Highest education qualification *(postgraduate degree, bachelor’s degree, diploma/trade certificate, completed high school, did not complete high school)*

**Baseline questionnaire**

*(Yes, to a large degree; moderately; neutral; somewhat; no, not at all)*

1. Awareness: Have you heard of monitoring, evaluation, and learning (MEL), or related terms, such as research or evaluation?
2. Beliefs: Do you believe MEL is important for improving participant/service user care quality or outcomes?
3. Attitudes: Are you willing to support MEL implementation?
4. Knowledge: Do you have sufficient knowledge to implement MEL principles?
5. Skills: Do you possess sufficient skills to implement MEL principles?

*(Yes; not sure; no)*

1. Implementation: In the past six-months, have you searched for relevant evidence in the literature (including best practice guidelines) to resolve questions related to your practice, and then applied the findings to decision-making after critical appraisal?

**Follow-up questionnaire (in addition to the above baseline questionnaire)**

1. Have you changed your practice decision-making through MEL implementation? *(Yes; not sure; no)*
2. Did you experience any of the below as being barriers to MEL implementation?

*Personal*

- Time, due to heavy work load
- Basic knowledge
- Skills in critical appraisal
- Skills in literature (or best practice) searching
- Incorporation with practice-focussed work
- Other

*Environmental*

- Library resources
- Capable designated personnel
- Access to MEL resources
- A MEL-supportive organisational culture
- Support from superior
- Other
