# Supplementary material for: Evaluation capacity building in a rural Victorian community service organisation: A formative evaluation
Source: PLoS One. 2025 Jul 30;20(7):e0322906. doi: 10.1371/journal.pone.0322906 (PMC12310013; doi:10.1371/journal.pone.0322906)
Supplement: S2 File — (DOCX) [file pone.0322906.s003.docx]

*Supplementary Material 3. Semi-structured interview guide.*

Hi, my name is [name of interviewer]. *Introduce self and other team members in the room, if applicable.*

*Introduce the project and explain the purpose of the interview*

As you know, the purpose of this interview is to understand your experience of implementing MEL at Brophy. This includes understanding what MEL has been like for you, what its impact was, and what needs to be improved going forward so that MEL can be expanded into other areas at Brophy. Your input will help us to evaluate the MEL implementation process so that we can maximize success going forward. This interview will be part of a formal evaluation, which we hope to publish in a peer-reviewed journal.

*Describe the audio-recording, confidentiality, and answer any questions*

With your permission, this interview will be audio-recorded so that we can have accurate records. The recordings will be kept confidential and will not be shared. Once your interview has been transcribed, a participant identifier will be assigned to each transcript so that you cannot be linked to it by anyone except the research team. You may request a copy of the transcription of your interview.

All your answers will remain confidential, so please refrain from using names during the interview. You may choose not to answer any of the interview questions. Your participation is voluntary, and you may withdraw your consent at any time without it affecting your work at Brophy. This study has ethics approval from Deakin University.

Do you have any questions before we begin? [answer any questions]

Are you ready to get started? [begin recording]

**Background**

I would like to ask you a few questions to help me understand your role within the MEL pilot.

1. Will you please describe your role, and how you were involved with the MEL pilot?

**Pre-implementation experiences**

1. Did you see a need to implement MEL? Why or why not?

*Probes*

- *What were the circumstances of MEL prior to the implementation of the pilot?*

**Implementation experiences**

1. What were the strategies you used to implement MEL?

*Probes*

- *How did you go about getting MEL in place?*
- *Were there stakeholders that you had to consult with to implement MEL?*

1. How does MEL fit within existing work processes and practices in your setting?

*Probes*

- *What were issues or complications that arose?*

1. Have you heard about the experiences of staff or clients with MEL?

*Probes*

- *Have you had any feedback from staff or clients about data collection?*

1. Has MEL been implemented according to the implementation plan?

*Probes*

- *How important do you think it is to implement MEL compared to the other priorities?*

1. What is required to continue MEL for this program/division going forward?

*Probes*

- *Are there any further resources or training requirements needed to help continue with MEL?*

**Impact**

1. Can you describe any changes to work practices which have occurred because of MEL?

*Probes*

- *Are there any work practices that have been amended because of the MEL pilot?*

1. How did you become upskilled during the MEL implementation?

*Probes*

- *What, if any, new skills did you learn?*

**Other**

1. Is there anything further you would like to add?

Thank you for your time and involvement with the MEL implementation.
